# Developing a master of science in health research ethics program in Northern Nigeria: a needs assessment

**DOI:** 10.1186/s12910-025-01165-w

**Published:** 2025-02-08

**Authors:** Caitlin Bieniek, Fatimah I. Tsiga-Ahmed, Aishatu L. Adamu, Usman J. Wudil, C. William Wester, Zubairu Iliyasu, Muktar H. Aliyu, Elisa J. Gordon, Elizabeth S. Rose

**Affiliations:** 1 Vanderbilt University, Nashville, USA; 2 Bayero University Kano, Kano, Nigeria; 3 Vanderbilt University Medical Center, Nashville, USA

**Keywords:** Health Research Ethics, Capacity building, Graduate Program, Education opportunities, Africa

## Abstract

**Background:**

Nigeria is an emerging hub of biomedical research, requiring additional trained bioethicists for ethical oversight of research studies. There are currently two graduate-level health research ethics programs in Nigeria. However, both are in the southern part of the country and no such training programs exist in the north. Strengthening the health research ethics skills and knowledge of Nigerian researchers across the country is necessary given the growing genetics research infrastructure.

**Methods:**

To inform the creation of a Master of Science in Health Research Ethics program in northern Nigeria, we conducted a needs assessment comprised of semi-structured interviews with nine Nigerian bioethics experts. We used the Interpretative Phenomenological Analysis (IPA) method to analyze interview transcriptions. Two authors independently read and coded each respondent’s transcript to identify emergent themes that represented each respondent’s answers. Within these overarching themes, the data points were grouped into subthemes.

**Results:**

Four primary themes emerged with ten subthemes. Respondents believed that the program can fill a gap and strengthen capacity in health research ethics. They emphasized that the curriculum should be developed with an interdisciplinary lens and locally contextualized, and that students should be taught how to think critically through ethical scenarios. Respondents stressed that program leaders should recruit faculty and students locally who have the bandwidth to participate in the program. Finally, respondents noted the program should have university support to be sustainable.

**Conclusion:**

Our findings will guide the creation of a master’s degree program that aims to build capacity in health research ethics in northern Nigeria and enhance the country’s growing prominence in global biomedical research. Through our needs assessment, we identified structural and content factors that can guide us in leveraging the strengths of the local institution and leaders in health research ethics while mitigating challenges in establishing this program.

**Supplementary Information:**

The online version contains supplementary material available at 10.1186/s12910-025-01165-w.

## Introduction

Nigeria is a growing center of scientific research, especially in the field of genomics and genetics. Strengthening the research ethics skills and knowledge of Nigerian researchers is necessary given the growing genetics research infrastructure. Since the late 1990s, scholars have increasingly recognized the need for ethical oversight of medical research in the country. A catalyst for this change was the 1996 “Trovan scandal” in which Pfizer, a pharmaceutical company, evaluated a new antibiotic, trovafloxacin (Trovan), during a large outbreak of meningococcal meningitis in Kano, northern Nigeria [1]. Multiple ethical lapses occurred, including failure to secure institutional review board (IRB) approval prior to the trial and not providing all patients with the same information about potential harm [2]. This incident highlighted the need for strengthening bioethics oversight of research across the region. There are currently two Master of Science (MSc) in research ethics programs in southern Nigeria, leaving a gap in research ethics training in the north. In northern Nigeria, there are more than 30 research institutions that could benefit from a local health research ethics program to prevent unethical research, like the Trovan scandal, from happening in the future. Most health research is conducted in clinical trials with very few basic (bench or animal) science studies and with few ethics review committees, proposal reviews take many months to a year to process. Hence, the need for health research ethics training remains essential in northern Nigeria, especially in the emerging area of genetics and genomics research.

Genetics and genomics research require ethical frameworks to guide research regarding the creation of specimen repositories, maintaining privacy, and informed consent [3]. Public knowledge in Nigeria about participants’ rights in medical and pharmaceutical clinical trials has been low [3]. This lack of knowledge can contribute to study participant mistrust and/or dissatisfaction, and the potential for ethical lapses [4]. Studies show that research ethics training programs for physician investigators can improve study participants’ understanding of clinical trials as well as the patient-physician relationship [3]. 

Historically, bioethics research funding has come from high-income countries enabling them to set research priorities [5]. To strengthen local research ethics capacity and to fill a regional gap in health research ethics knowledge, leaders at a university in northern Nigeria proposed creating an MSc program in Health Research Ethics. We conducted a needs assessment to better understand the needs, gaps, opportunities, and potential challenges in developing the degree program. With a deeper understanding of considerations for program implementation, we hope to maximize the effectiveness of this MSc program.

## Methods

### Study design

This needs assessment aimed to understand local strengths and potential challenges in the creation of an MSc program that would strengthen the cadre of Nigerian researchers with ethics expertise to support the expansion of genomics research in the country. We conducted a cross-sectional qualitative study in April 2024 to elicit feedback on curriculum, courses, and program structure. We received ethical approval for the study and respondents provided verbal informed consent prior to participation. To maintain a conversational feeling, we did not have respondents provide written consent. Ethical approval was obtained from the university’s research ethics committee prior to the start of data collection. We used the Standards for Reporting Qualitative Research checklist for quality reporting [6]. 

### Subject eligibility and recruitment

We selected individuals who reside and work in Nigeria and who are prominent and qualified figures in academia, medicine, and research ethics. These individuals work at various universities, hospitals, and federal offices across Nigeria, including Amino Kano Teaching Hospital, Bayero University Kano, the Federal Ministry of Health, and the National Health Research Ethics Committee. Each individual was selected because they are a stakeholder in the creation of the program as they have the potential to be professors, mentors, colleagues, and employers of future students and graduates. The respondents provided insights about optimal approaches for designing research ethics training programs based on their experience working in their various fields. All respondents were professional associates of the authors and were emailed an invitation to participate in an interview.

### Data collection

We conducted nine individual semi-structured interviews using an online video conferencing platform, Microsoft Office Teams. To protect the confidentiality and privacy of our respondents, we did not record the interviews. The two authors who conducted the interviews each took notes throughout then wrote summary reports. We developed and used an interview guide, comprised of ten questions and sixteen probes about the strengths of and opportunities for research ethics and associated training in Nigeria, potential challenges implementing an MSc program, and suggestions for the curriculum, faculty, and students (Supplemental File 1). The interview guide was created for this needs assessment and the questions were tailored to the specific university in Nigeria. The questions in the interview guide were based on the authors’ previous experience of what makes for good needs assessment as well as genuine questions that we had regarding the creation of the program [7, 8]. We used our research aims to guide the development of the interview guide and used the “Low-Burden Needs Assessment Questions” to inform the interview questions [9]. We conducted interviews until we reached data saturation. Each interview lasted about one hour.

### Qualitative analysis

We used the Interpretative Phenomenological Analysis (IPA) method to analyze interview responses [10, 11]. IPA is a qualitative research methodology in which researchers collect participants’ stories to understand how they make sense of experiences [10]. Central to IPA is a double hermeneutic process in which researchers guide participants through “sense-making” of their experiences in an interview and then the researcher interprets the participants’ sense-making [11]. Small, homogenous samples with four to ten participants are generally used in IPA studies [12, 13]. Researchers use deductive and inductive practices to design the study and analyze findings [10, 14]. 

Two authors independently read each respondent’s answers to identify emergent themes. Then, they independently and manually created a one- or two-word code for each theme, which were put on a shared Microsoft Excel Sheet. Afterward, they discussed any discrepancies in their coding to reach a consensus. The authors then collated emergent themes to identify connections and patterns across respondents’ responses that highlighted overarching themes. Within these overarching themes, the data points were grouped into subthemes. The authors’ summaries of the interview documents and themes data Excel sheet were kept in shared Microsoft Office folder. All study documents were stored in an encrypted SharePoint folder only accessible by the authors.

## Results

All nine recruited individuals completed an interview (100% participation rate). The respondents have currently or in the recent past held multiple roles in medical education and research, including as educators (*n* = 7), researchers (6), clinicians (4), members of ethics committees (4), and ethics graduate students (2). We identified four overarching themes with ten subthemes (Table 1).

**Table 1:** Summary of the four themes that were drawn from interview responses

Theme	Subthemes	Description
**Opportunity for Strengthening Capacity**	• Bioethics is a growing field in Nigeria • Job opportunities	This program can strengthen capacity in a growing field in Nigeria. The program will help students advance their careers in bioethics.
**Curriculum and Program Structure**	• Local perspectives • Skills-based • Interdisciplinary lens • Dissertation • Hybrid and flexible program	There were many suggestions regarding what the course work and program should entail. Most emphasized interdisciplinary topics and enhancing practical skills of the students.
**Qualities of Potential Faculty and Students**	• Recruitment	Recruiting dedicated students and faculty is necessary to ensure a successful program.
**Programmatic and Institutional Support**	• Physical resources and funding • Administrative support	Buy-in from the University is crucial to secure the program’s future. The program should provide certain resources for students.

### Theme one: opportunity for strengthening capacity in Health Research Ethics

All respondents expressed enthusiasm about the potential program filling a training gap in northern Nigeria and optimism for the program’s success. Respondents pointed out the significant cultural and religious differences between the northern and southern regions of the country, and that despite the large population and numerous universities and teaching hospitals in the north, that region lacks graduate training in health research ethics. People in northern Nigeria are majority Muslim and have historically been distrustful of western practices that are more common in the southern part of the country where Christianity is more prevalent [15]. Hence, they indicated a significant need for a training program in the north that would be culturally appropriate for the region.

Nearly all noted that bioethics and research in genomics and genetics are expanding fields. However, respondents recognized that health research ethics is still in an “*infancy stage*” (Respondent two) in Nigeria. Respondent four reported that “*not many people have embraced the idea of research ethics or bioethics*” and that “*it’s important to help people understand what ethics is and to put it into practice*.” S/he described that the processes around research ethics approval are “*not well understood*” and that some researchers “*see ethics as a block in research*” since approval processes can be costly and delay the start of a study. Thus, the master’s program will be tasked with increasing awareness of the need for research ethics and associated approval processes. However, Respondent eight stated that medical and nursing students who are already doing research have some knowledge of research ethics, particularly regarding informed consent. Respondents suggested that through courses, case studies, and practical observations, the proposed master’s program will help expand and formalize that knowledge by educating a new generation of researchers.

Some respondents discussed that program graduates will have a variety of options for career growth. A master’s degree will give trainees a competitive edge in advancing their careers in this expanding field. Graduates would be qualified to work on local and national institutional review boards (ethics review committees) or in international health organizations, such as the Africa Centers for Disease Control and Prevention. The program should also be complementary to students’ current work and expand their capacity in that role. Respondent nine suggested the proposed program could aid in improving physician-patient relationships. By providing training in ethics, the practice of clinical ethics could be improved by physicians providing patients with a comprehensive informed consent process. This example highlighted program opportunities and the need for ethics in real world circumstances and case studies, rather than focusing on learning theory without application.

### Theme two: curriculum and program structure

Respondents emphasized that the curriculum should incorporate perspectives local to Nigeria and the African continent. They discussed that experiences and philosophical perspectives in Kano are different from other global regions, but research ethics curricula commonly reflect Western values and beliefs that are grounded in individualism. For example, Respondent four commented that local views around palliative and end-of-life care differ from Western views, and these perspectives need to be considered when teaching ethics. Curricular components such as course content, case studies, and other elements should reflect regional beliefs, perspectives, religion, and culture. Respondent two discussed the need to involve community members involved in bioethics to participate in the creation of courses or participate as guest lecturers, which would help in contextualizing the program.

When asked about elements of an ideal master’s degree program, all respondents described an interdisciplinary curriculum that teaches practical skills in assessing ethical situations, rather than being solely theoretical. They explained that because ethics is a process and not a memorization of theories, students should be taught how to think critically through challenging situations. Specific skills-building courses and topics they suggested included research methods, data analysis, biostatistics, grant writing, and presenting results. Respondents recommended that courses cover topics spanning multiple disciplines to ensure students are prepared to address ethical situations across research areas and medical specialties. For example, the curriculum should incorporate courses from the humanities and public health taught with a bioethics lens. Additionally, Respondent five recommended that the curriculum development process should begin with a meeting with representation by at least six disciplines including law, social sciences, psychology, and health.

Five respondents discussed the student’s ‘dissertation’ (referred to as a master’s ‘thesis’ in other countries), a commonly shared feature of master’s programs that students usually prepare in the second year of a two-year master’s degree program in Nigeria. Respondents described challenges that students have encountered in other institutions in preparing their dissertations, including working full-time, a lack of dissertation supervisors, and complex administrative processes. Respondent seven recommended that students should start working on their dissertation early in the program, rather than waiting until the second year, to maximize time to work on their research. They suggested that students incorporate ideas about their dissertations into their course work in the first year (e.g., conduct a literature review for a class assignment), which would give them more time to study their topic and prepare for research.

A challenge other MSc programs have experienced is a lack of faculty bandwidth to mentor students in their dissertations. Faculty support and mentorship are instrumental for students to have a fulfilling experience. To address issues with faculty bandwidth, Respondent four suggested that students partner with a faculty member on that faculty member’s ongoing research instead of creating a research project *de novo*. Respondent three proposed that students could work in groups on dissertation research, which could address bandwidth issues and help students develop important teamwork skills, since post-graduation work is commonly conducted in teams. Respondent five suggested that students present their dissertation abstracts at annual national ethics conferences as a milestone and form of motivation. Some respondents described administrative challenges with scheduling dates for the proposal, internal, and external defenses, which can delay graduation, and suggested alternative formats for students to present their work or to take exams that may pose fewer scheduling issues.

Respondents recommended that the master’s program be “accessible” (i.e., be flexible) for students who work full-time and/or have family commitments. Respondents noted that students in the other Nigerian ethics master’s programs are often full-time working professionals who take a sabbatical to attend classes and return to working full-time during the dissertation phase. However, work obligations limit their time bandwidth to engage in research and writing, which slows their dissertation progress and delays their eventual graduation.

Three respondents recommended a hybrid format with a mix of courses taught by the instructor in-person or virtually. For example, the first semester (six months) of the program could be taught in-person with the remainder of courses and program elements held remotely. Respondent seven suggested including supplemental online courses and webinars. However, everyone underscored the importance of in-person elements, with practical experiences, such as observing an ethics review committee, being the most common component that should be in-person.

Respondents described the importance of building practical skills through practical experiences such as observing ethics review committees and shadowing clinicians. They noted that these experiences should be selected carefully so that students have a formative experience and build requisite skills. Respondents varied in sentiments about whether these practical experiences should be a required formal practicum (i.e., a mentored experience in which students apply knowledge learned in the classroom).

### Theme three: qualities of potential Faculty and Students

Respondents shared ideas about qualities for potential faculty and students and where to recruit them. Faculty should be experts who can teach and are ready (willing) to teach. For example, Nigerian faculty could be recruited from research ethics committees or other ethics training programs. Respondents suggested that the faculty must have training in mentorship, which was described as an area that could be enhanced.

Two respondents advocated that students must be recruited from across disciplines. They recommended prioritizing students from northern Nigeria, where there is a lack of research ethics training programs, recruiting them from teaching hospitals, research ethics review committees, and other institutions in Kano and the surrounding areas. Program leaders should select students who are motivated and able to commit to completing the program in two years rather than “*doing the degree on the side as something extra*” as Respondent six noted.

### Theme four: programmatic and institutional support

Respondents highlighted that the program and local partner institutions should support students by providing physical resources and funding, faculty mentorship, and administrative support. Respondent three noted that providing students with access to physical resources, including bibliographic literature, both in print and digital, quantitative and qualitative data analysis software packages (e.g., Stata, SPSS, NVivo), Wi-Fi, and accommodations (e.g., housing), is important to help students thrive. Underlying their discussion of resources was the possibility that students might not have the financial means to afford such resources and may be unable to afford tuition without assistance. Some questioned how funding will be sustained beyond the grant period to continue supporting students’ tuition and other fees. Program leaders will need to find creative solutions to support program growth and sustainability.

Three respondents stated that before starting a program, obtaining buy-in from the university will be important for helping students feel supported throughout their program. University support could also help ease bottlenecks associated with administrative processes (e.g., scheduling the multiple dates for the proposal, internal, and external defenses) that, according to Respondent seven, delayed dissertation completion and graduation in other programs.

## Discussion

All respondents, who collectively represent leaders, researchers, and educators in research ethics across Nigeria, expressed enthusiasm for the proposed master’s program and uniformly agreed that it would fill a regional gap in bioethics research training. Additional key findings pertained to creating an interdisciplinary and local curriculum, recruiting local faculty and students with appropriate bandwidth, and university support for the program. Across the interviews, we found that respondents were in consensus about several topics, resulting in multiple overlapping themes.

The recommendations on program structure, need for flexibility, and ways to improve the dissertation process support the need for the program to be relevant to students and the health research ethics field. The program should provide a supportive environment to learn as well as knowledge and tools that will be useful post-graduation. A large meta-analysis found that ethics courses can be delivered using a hybrid approach that incorporates a blend of instructional formats and content areas [15]. As suggested by respondents, program leaders will need to consider innovative suggestions regarding an interdisciplinary curriculum, hybrid program formats, and the dissertation process.

The program should be interdisciplinary and train students in practical skills. These suggestions reflected a need for students to apply skills where there is a gap between professionals and work across disciplines. This recommendation aligns with the evidence that ethics training courses are more effective when designed to improve practical skills rather than changing attitudes about ethics issues [15–17]. 

This program should recruit faculty and students from various disciplines and medical specialties. Recruiting interdisciplinary students and faculty will prepare students to address ethical issues across different situations in clinical trials or medical research. Another study found that nurses with formal ethics training performed better on posttest questionnaires about best ethical practices compared to nurses who did not, and the ethics-trained nurses applied the training to improving care for their patients [18]. Faculty with non-medical training are necessary to teach courses, and students with diverse professional backgrounds will help make class discussions robust with varying experiences. In advertising the program, efforts should be directed to raise awareness of the program so that it reaches individuals who are ready and willing to pursue careers in health research ethics and who have time to dedicate to the program.

Interviews associated with H3Africa, a genomics and biobanking initiative across the continent, voiced concerns with program sustainability since it was funded externally, and they recommended involving local stakeholders in the development of the program [19]. A similar concern was noted by our respondents as they described the necessity of university support for the program.

Along with the need to garner university support, respondents expressed local faculty members should be an essential part of curriculum and course development. Including local perspectives in the curriculum development and ensuring university support (i.e., so the program is sustainable without foreign financial support) highlighted the issue of external organizations imposing the use of their curricula without contextualizing the content or ensuring that the program is needed [20]. This need to place local faculty at the forefront aligns with Fayemi and Macaulay-Adeyelure’s [21] recommendations for decolonizing bioethics in Africa that local program leaders and faculty should have control over the curriculum to provide ethical lens from their experiences and communities [21]. 

Local program leaders and faculty will need to remain at the forefront of discussions and planning regarding the program structure and curriculum. Nigerian experts in ethics, such as those interviewed for this needs assessment, should be consulted about course development and other curricular components. Courses should be developed by the university faculty using regional case studies and in line with local cultural values. For example, a local philosophical perspective is called *Ubuntu* (“I am because we are”). *Ubuntu* is an African perspective based on the concept of holism and humanism [22]. This community-focused philosophy emphasizes respect for life and positive community relationships [22]. 

Effective mentoring relationships are important for building students’ knowledge and skills and enabling the development of professional relationships that positively impact trainees’ career trajectories [23]. However, mentors (e.g., dissertation supervisors) must have the bandwidth to work with students; identifying ways to better enable faculty with limited bandwidth to engage in mentorship is urgently needed. The inclusion of a carefully crafted interdisciplinary, collaborative, and mentee-driven mentoring model that is not solely reliant on program faculty can be used to train students to assume active roles in the mentorship process and provide opportunities for students to learn from experts beyond the classroom [24]. Since the bioethics research field in Nigeria is growing and there are limited local experts, program alumni with enhanced practical skills may be well positioned to help guide students, the field, and standards of practice. The proposed training program has the potential to shape the workforce and perspectives of bioethics research in Nigeria.

### Trustworthiness and limitations of findings

Demonstrating trustworthiness in qualitative research is essential [25, 26]. Researchers can show trustworthiness through the elements of credibility, transferability, dependability, and confirmability. A strength of this study was our ability to obtain valuable in-depth data from ethics experts using a validated qualitative methodology approach, Interpretative Phenomenological Analysis, which enhances the credibility of our report. At the beginning of each interview, we built a rapport with the respondent, which demonstrates the credibility of the information we gathered. To ensure that our analysis and results were dependable, we kept detailed notes and summaries of all our interviews. We documented the themes identified so that our interpretations of responses were closely aligned with respondents’ sentiments. Finally, in establishing confirmability, we acknowledge that our biases may have influenced the interpretation and analysis of interview responses. We sought to mitigate the impact of our biases through bracketing (a qualitative technique) during individual review and then used peer debriefing to review our interpretations and findings.

We conducted interviews on online platforms, which may have diminished non-verbal body cues discernable with face-to-face encounters. In addition, Wi-Fi connections were not always stable, leading to occasional missed phrases, turning off video for better connectivity, or rescheduling. Another limitation is that most respondents were men. More perspectives from women may have provided additional insights. Additionally, the respondents are stakeholders in this potential program, which may have biased their responses. However, we selected these professionals because they are some of the most equipped people to answer questions about improving health research ethics training in Nigeria. While limiting, our selection is also a strength of this study.

## Conclusion

Through our needs assessment, we identified structural and content factors that can guide us in leveraging the strengths of the local institution and leaders in research ethics while mitigating challenges in establishing this master’s program. Program development and capacity building take time, and our findings revealed a strong interest in seeing this program succeed. As we work to increase research ethics capacity to support the expansion of medical research in Nigeria, we can learn from the experiences of other research ethics programs to create one that is complementary but unique. Through the proposed MSc program, Nigerian researchers will gain the necessary skills and knowledge to advance the field of research ethics. With dedicated local program leaders and an interdisciplinary curriculum contextualized to the region, this program can help fill the need for trained bioethics specialists to serve on research ethics committees and in other capacities to help ensure the ethical conduct of research in northern Nigeria and beyond.

## Electronic supplementary material

Below is the link to the electronic supplementary material.

Supplementary Material 1

## Data Availability

Data is available upon request. If you would like the data, please contact the first author Caitlin Bieniek or Dr. Elizabeth Rose at Vanderbilt Institute of Global Health.
