# Supplementary material for: Developing a master of science in health research ethics program in Northern Nigeria: a needs assessment
Source: BMC Med Ethics. 2025 Feb 8;26:24. doi: 10.1186/s12910-025-01165-w (PMC11806612; doi:10.1186/s12910-025-01165-w)
Supplement: Supplementary file 1 — Supplementary Material 1 [file 12910_2025_1165_MOESM1_ESM.docx]

**Needs Assessment Semi-Structured Interview Questions**

**Master of Science in Research Ethics, Bayero University Kano**

**Question guide**

- Please describe your role in research ethics in Nigeria
- Please describe the current environment for research ethics and bioethics at BUK/AKTH, in Kano, and in Nigeria.
- *What are areas of strength? Who are the local experts and organizations?*
- *What are areas that could be improved? Are there knowledge and skills that need to be bolstered?*
- Please describe skills and knowledge that are needed for current and future researchers in research ethics and bioethics.
- *Please describe ways that courses could equip students with this ethics knowledge and skills.*
- *Please describe any suggestions for specific course topics and elements.*
- *Please name any individuals who might be well-suited and interested in teaching these courses.*
- *Please* *describe* *local and regional histories and perspectives that should be incorporated into courses and the curriculum.*
- *Please describe dissertation mentors that might be open to mentoring a student.*
- *Please describe potential dissertation projects that would help students build skills and fill a research gap.*
- *Please describe any opportunities for practical experience at BUK/AKTH, in Kano, and in the region.*
- *Please describe other resources (e.g., local organizations, online courses, courses at other institutions) that would be useful for students.*
- Please describe potential post graduate job opportunities locally and regionally.
- Please describe potential challenges and obstacles that we might encounter in starting and maintaining the program.
- *What needs to be addressed before beginning the program?*
- *Are there university and local politics should we be aware of regarding program development and implementation?*
- *Are there legislative policies (present or future) that could help or harm the program?*
- (For other program leaders) Please describe lessons learned from developing other programs.
- Who are the key people that should be involved in the planning and implementation processes?
- *Who are key people who should be informed about our work during the planning and implementation phases?*
- (For people at BUK) What campus resources are available to support the program and students?
- *i.e. for students: internet access, library access, other support services, ethics committee, mentors*
- *i.e. for the program: administrative staff, classroom space, available faculty, relevant classes that could be cross listed*
- What would perfect implementation of the program look like?
- What other questions should I ask?

**Semi-structured interview guide: For investigators at BUK**

*Curriculum*

- Courses
- This degree program will be 32 credit hours – how many courses is that?
- It looks like we can use the two research ethics courses from the MScPH (Medical Ethics & Law; Global Health Ethics & Law; 2 credits each; taught by Prof Iliyasu & Dr Umar). Is there capacity in these courses for additional students?
- The bioethics curriculum includes 10 courses. How many of those courses could we cross-count for our program? Is there space in these courses for additional students? If not, could additional sections of those courses be taught by the same professor or by a different professor?
- In the grant proposal, we proposed creating the following courses (see table). Are there faculty who have been identified to develop and teach these courses? Are there resources that we can use in developing these courses?

| *Introduction to Ethical Theory:* systematically introduces ethical theory, including analyses of utilitarianism, justice, responsibility, the meaning of ethical terms, and relativism. |
| --- |
| *Foundations of Bioethics:* examines the moral frameworks individuals use in ethics decision-making and introduces students to the conceptual framework specific to the field of bioethics. |
| *Introduction to Research Ethics:* covers basic principles, guidelines, and policies relating to ethical research. |
| *Introduction to Data Ethics:* introduces concepts of data privacy, intellectual ownership of samples, and data sharing |
| *Introduction to Bioethical Argument:* examines how bioethical arguments are constructed with the objective of mastering both the critique of bioethical arguments and their construction. |
| *Ethics, Genetics, and Genomics:* explores key ethical issues relating to the emerging field of genomics and personalized medicine. |
| *Institutional Review Boards:* covers essential information and best practices for IRB administration. |
| *Seminars in Bioethics:* explores ethical questions raised by emerging genomics technology, clinical trials in LMIC settings, drug development, pandemic response policies, and other topics. |
| *Genomic Research Ethics Journal Club:* creates a space to critique and appraise evidence-based genetic/genomic research and discuss ethical dilemmas related to genetic and genomic research. |
| *Responsible Conduct of Research (RCR) in International Settings*: provides an overview of the key issues surrounding the ethical conduct of research in LMIC settings, including rigor and reproducibility, authorship, conflicts of interest, etc |

- Faculty
- What other faculty members teach research ethics?
- Would they be able and open to creating and teaching additional courses?
- What other faculty members have experience in research ethics and could develop and/or teach courses in this program?
- What courses have been taught previously that could be used for this program?
- What are local and regional histories and perspectives that should be incorporated into courses and the curriculum?

- Curriculum structure
- Does the thesis count towards the 32 credit hours?
- What is the general process, timeline, and structure for a thesis at BUK? (i.e., do students work independently, how are students mentored, is there a formal/informal course, how do they select a topic, when do they conduct their research [concurrent with courses or protected time], etc.)
- Are there other requirements (e.g., practical experience, service, comprehensive exam, etc.) that need to be included in the curriculum?

- Instructional modalities
- What practices are currently used for teaching? Which practices would be useful in this program? E.g., lecture, case-based, small group discussion, small group projects, etc.
- Have distance learning modules been used in courses? Could those be incorporated to include experts who are outside Kano?

*Resources*

- What are BUK/AKTH’s strengths in research ethics? (e.g., faculty, staff, review committees, awards)
- Who are the leaders in research ethics in Nigeria, West Africa, and Africa?
- How might we involve them in the program? (e.g., guest lecturers, remotely teach a course, mentor students, provide practical experiences, etc.)
- What opportunities are there for thesis research at BUK/AKTH, in Kano, and in the region?
- What campus resources are available to support the program and students?
- i.e. for students: internet access, library access, other support services, ethics committee, mentors
- i.e. for the program: administrative staff, classroom space, available faculty, relevant classes that could be cross listed

*Program implementation*

- What potential opportunities and challenges might we encounter starting the program?
- Are there issues that must be addressed before the program can begin?
- Are there university and/or local politics that we should be aware of regarding program development and implementation?
- What policies (present or future) could help or harm the program?
- Who are the key people that should be *involved* in the planning and implementation processes? Who are key people who should be *informed* about our work during the planning and implementation phases?
- Please describe the process and timeline for applying for university approval for a new degree program.
- What can we learn from recently established degree programs (e.g., Maternal and Child Health, Public Health Nursing, Public Mental Health, and Public Health Nutrition)?
- What is the process and timeline for accreditation with the Nigerian National Universities Commission?
- How will the program be funded after the grant ends? What potential challenges and obstacles might we encounter in ensuring the program’s sustainability?

*Experience of Other Programs*

- What might we learn from the master’s in ethics degree program at the University of Nigeria (Dr. Ezeome)?
- Could we access curricular materials?
- Other programs we should consider/reach out to?
- What has been the student experience? What can we learn?

*Sustainability*

- After the grant period ends, who are the potential students who would be interested (and enroll) in this program?
- How will the program be advertised?
- How will the program follow fair recruitment and admission practices?
- How will the program sustain enrollment after the grant period ends (i.e., will students matriculate without scholarship support)?
- What post-graduation job opportunities might be available for students both locally and regionally?

*Concluding questions*

- What would perfect implementation of the program look like?
- What questions should I have asked that I haven’t?
